# PW01-008 – The inflammasome and secretory pathways in FMF

**DOI:** 10.1186/1546-0096-11-S1-A61

**Published:** 2013-11-08

**Authors:** B Orak, T Kallinich, M Lieber, H von Bernuth, H Wittkowski, D Foell

**Affiliations:** 1 Paediatric Rheumatology, Charite University Medicine Berlin, Germany; 2 Paediatric Rheumatology and Immunology, Charite University Medicine Berlin, Berlin, Germany; 3 Paediatric Rheumatology, University Hospital of Muenster, Germany; 4 Paediatric Rheumatology, University Hospital of Muenster, Muenster, Germany

## Introduction

Aberrant inflammasome priming and dysregulated secretory pathways contribute to parallel IL-18 and S100A12 hypersecretion from neutrophils in instable FMF.

## Objectives

The study was performed to assess the ex vivo inflammasome activity in granulocytes from patients with unstable FMF. Furthermore, the secretion of S100A12 molecules after various kinds of cell stimulation was assessed.

## Methods

6 turkish patients with the clinical diagnosis of FMF exhibiting homocygous or combined heterozygous mutations within the MEFV gene were included. Patients still exhibited clinical symptoms and elevated inflammation markers despite sufficient colchicine therapy (“instable disease”). Healthy probands served as controls. Their health status was assessed by a standardized questionnaire. All patients and controls gave written consent.

25 – 30 ml blood was drawn and PBMC and granulocytes separated by a two density gradient centrifugation. 5 x 106 cells were stimulated with (i) mock, (ii) PMA (10nM), (iii) LPS (10ng/ml) and (iv) LPS (10ng/ml) + ATP (1mM) (later substance for the last 30 minutes). In a similar approach cells were treated with additional colchicine (5μg/ml) for the whole incubation time. Supernatant was gained and frozen at -20oC after 5 hours. ELISA for S100A12, IL-18 and caspase-1 were performed according to standard protocols.

At the time of blood drawing high sensitivity CRP was measured.

## Results

Compared to controls even unstimulated granuloytes from FMF patients with instable disease secreted significantly more S100A12 (mean controls 43ng/ml vs. mean patients 327ng/ml, p <.01), IL-18 (0pg/ml vs. 274pg/ml, p <.01) and caspase-1 (10pg/ml vs. 81pg/ml, p <.01). Stimulation also induced enhanced secretion of S100A12 (PMA: 61ng/ml vs. 336ng/ml, p <.01; LPS: 74ng/ml vs. 247ng/ml, p <.01; LPS/ATP: 94ng/ml vs. 252ng/ml, p <.01), IL-18 (LPS: 3,6pg/ml vs 176pg/ml, p <.05; LPS/ATP: 7pg/ml vs. 198pg/ml, p <.05) and caspase-1 (LPS: 23pg/ml vs. 72pg/ml, p <.01; LPS/ATP: 32pg/ml vs. 69pg/ml, p <.05). Furthermore, supplementary colchicine significantly suppressed the hypersecretion of S100A12, IL-18 and caspase-1.

## Conclusion

The spontaneous release of IL-18 and caspase-1 demonstrated the constant inflammasome activity in patients with instable FMF. It was not be further induced by LPS/ATP stimulation. S100A12, although not processed by the inflammasome, was also secreted in high amount from FMF neutrophils, irrespective of further stimulation. Taken together, these data indicate that FMF neutrophils show a spontaneous hypersecretion of S100A12 and IL-18 which is only partially related to aberrant inflammasome activation (shown by parallel Caspase-1 release) but may also dependent on dysregulated alternative secretion (inhibited by colchicine in vitro).

## Disclosure of interest

None declared

